# On the Examination of Ointments Containing Oxide of Mercury

**Published:** 1852-09

**Authors:** 


*On the Examination of Ointments containing Oxide of Mercury.* By M. Robierre.—Having had occasion to examine some citrine ointments employed by a woman who was accused of illegally practising medicine, I experienced some difficulty in ascertaining the chemical characters of the metallic substance present, in consequence of the small proportion of it contained in the ointment, and also in consequence of the ointment being very old. The following method of operating proved so successful, that I am induced to record it for the benefit of those engaged in such investigations. It serves to isolate mercury in a few minutes from its combination with oxygen and fatty acids. However small the quantity of mercury present may be, the effect is, nevertheless, distinct.

The ointment to be examined is melted by the application of a gentle heat, and a small quantity of the essence of citron is then added to it. Under the well-known reducing influence of this hydro-carbon, the ointment acquires a grey color, which effect is to be promoted by agitation. After about five minutes, the ointment being still kept melted, three times its volume of ether is to be added, the whole mixed together, and then allowed to stand. The supernatant liquid is then to be decanted, and the residue washed several times with ether. The mercury left at the bottom of the vessel may now be dissolved in nitric acid, and tested with the usual reagents.—*Journal de Chimie Médicale.*

